# Effects of mineralocorticoid receptor antagonists in patients with preserved ejection fraction: a meta-analysis of randomized clinical trials

**DOI:** 10.1186/s12916-014-0261-8

**Published:** 2015-01-19

**Authors:** Yanmei Chen, He Wang, Yongkang Lu, Xiaobo Huang, Yulin Liao, Jianping Bin

**Affiliations:** State Key Lab for Organ Failure Research, Department of Cardiology, Nanfang Hospital, Southern Medical University, 1838 North Guangzhou Avenue, Guangzhou, 510515 China

**Keywords:** Meta-analysis, Mineralocorticoid receptor antagonists, Preserved ejection fraction, Randomized controlled trial

## Abstract

**Background:**

Mineralocorticoid receptor antagonists (MRAs) have been shown to be effective in patients with heart failure or myocardial infarction complicated by a reduced ejection fraction. However, the role of MRAs in patients with preserved ejection fraction (PEF) remains to be clarified. We aimed to summarize the evidence for the efficacy of MRAs in patients with either heart failure with PEF (HF-PEF) or myocardial infarction with PEF (MI-PEF).

**Methods:**

We searched PubMed, EMBASE, Cochrane Library, and clinical trials databases for randomized controlled trials, through June 2014, assessing MRA treatment in HF-PEF or MI-PEF patients. Fourteen randomized controlled trials (MI-PEF, 5; HF-PEF, 9; n = 6,428 patients) were included.

**Results:**

MRA treatment reduced the risk of hospitalization for heart failure (relative risk, 0.83; 95% confidence interval [CI], 0.70 to 0.98), improved quality of life (weighted mean difference [WMD], −5.16; 95% CI, −8.03 to −2.30), left ventricular end-diastolic diameter (standardized mean difference, −0.21; 95% CI, 0.32 to −0.11), and serum amino-terminal peptide of procollagen type-III level (WMD, −1.50, 95% CI, −1.72 to −1.29) in patients with PEF. In addition, MRAs reduced E/e'(an echocardiographic estimate of filling pressure for assessment of diastolic function; WMD, −1.82; 95% CI, −2.23 to −1.42) in HF-PEF patients and E/A ratio (the ratio of early to late diastolic transmitral flow; WMD, 0.12; 95% CI, 0.10 to 0.14) in MI-PEF patients. However, all-cause mortality was not improved by MRAs in either HF-PEF (*P* = 0.90) or MI-PEF (*P* = 0.27) patients.

**Conclusions:**

MRA treatment in PEF patients led to reduced hospitalization for heart failure, quantifiable improvements in quality of life and diastolic function, and reversal of cardiac remodeling, but did not provide any all-cause mortality benefit.

**Electronic supplementary material:**

The online version of this article (doi:10.1186/s12916-014-0261-8) contains supplementary material, which is available to authorized users.

## Background

Approximately half of patients with heart failure (HF) have normal or only mildly impaired left ventricular ejection fractions (LVEFs) [1,2]. Patients with this profile, known as HF with preserved ejection fraction (HF-PEF), have signs, symptoms, quality of life (QoL), and prognoses similar to HF patients with a reduced ejection fraction (HF-REF) [3,4]. Furthermore, patients with acute myocardial infarction (MI) often have preserved ejection fraction (PEF) [5]. Although many medical therapies benefit HF patients and post-MI patients with reduced LVEF [6], effective, evidence-based pharmacologic treatments are not currently available for PEF patients [7].

Aldeosterone-based activation of mineralocorticoid receptors has been demonstrated to contribute to the pathogenesis of HF and adverse cardiac remodeling after MI through multiple mechanisms, mainly including sympathetic activation, promotion of cardiac and vascular fibrosis, endothelial dysfunction, sodium retention, and potassium loss [8,9]. Mineralocorticoid receptor antagonists (MRAs) may inhibit these deleterious effects [10] and may contribute to a beneficial therapeutic strategy for PEF patients. MRAs are effective for reducing total and cardiovascular mortality in patients with HF-REF (LVEF <35%) and post-MI patients with left ventricular dysfunction (LVEF <40%) [11-13]. However, whether they have a role in PEF remains to be clarified.

A recent series of studies assessed the efficacy of MRAs in HF-PEF patients and in patients with PEF after MI (MI-PEF) [14-19]. Although some studies failed to show a significant mortality benefit for MRA use [14,15], a number demonstrated a range of secondary benefits such as improved QoL, diastolic function, and cardiac remodeling, in response to MRA therapy [16-19]. As patients with PEF are usually older than HF-REF patients, a comprehensive evaluation may help provide support for therapy that improves symptoms and QoL, rather than mortality. In addition, since diastolic dysfunction and cardiac remodeling are considered the major underlying cardiac pathophysiology in HF-PEF and MI-PEF [20], combining data regarding the impact of MRAs on these related parameters might elucidate some encouraging findings. However, data combining the experience from published randomized controlled trials to evaluate the effects of MRAs in PEF patients do not exist. Given the limited evidence concerning MRAs in PEF patients, this meta-analysis aimed to summarize the available data from randomized controlled trials (RCTs) to determine the efficacy and safety of MRAs in PEF (including both HF-PEF and MI-PEF) patients.

## Methods

This meta-analysis was performed and reported according to the Preferred Reporting Items for Systematic Reviews and Meta-Analyses (PRISMA) guidelines (Additional file 1) [21].

### Literature search

We searched the MEDLINE, EMBASE, Cochrane Library databases, and clinical trials databases (clinicaltrials.gov, controlled-trials.com, and clinicaltrialsregister.eu) for randomized controlled trials conducted between January 2000 and June 2014, using the following key words: i) mineralocorticoid receptor antagonists, aldosterone receptor antagonist, canrenoate, canrenoate potassium, canrenone, canrenoic acid, spironolactone, or eplerenone; ii) preserved left ventricular function, preserved ejection fraction, heart failure with normal ejection fraction, or diastolic heart failure; and iii) randomized controlled trials. Our literature search was limited to studies involving human subjects, reported in English. The list of full search strategies for EMBASE and MEDLINE is provided in Additional file 2. The search strategies for other databases are available on request.

### Inclusion criteria

We included prospective, RCTs that: i) enrolled adult PEF patients with LVEFs ≥40% (including post-MI patients and those with symptomatic or asymptomatic HF), ii) assigned patients to MRA treatment versus placebo or control, iii) had at least one of the clinical outcomes of interest, and iv) had a study duration of at least 4 months.

### Data extraction

Two independent reviewers screened all titles and the abstracts of all citations; potentially relevant articles were assessed according to the inclusion criteria. Disagreements were discussed until a consensus on inclusion/exclusion was reached. Information on patient characteristics, study design, quality, intervention strategies, and clinical outcomes was systematically extracted from each report using a standardized form. Data regarding safety and adverse events, including hyperkalemia and gynecomastia, were also noted. Hyperkalemia was defined as a potassium level >5.5 mmol/L. We used definitions of renal failure and gynecomastia as per the primary trial publication. The quality of the included RCTs was assessed using the Jadad quality scale [22].

### Outcome measures

The clinical outcomes for this meta-analysis were all-cause mortality and hospitalization due to HF. We also assessed echocardiographic parameters related to diastolic function, including E/e' (an estimate of filling pressure used to assess diastolic function), E/A (the ratio of early to late diastolic transmitral flow), E-wave deceleration time, and isovolumic relaxation time (IVRT) andvariables related to left ventricular structure and function, including LVEF, left ventricular end-diastolic volume index, left atrial volume index (LAVI), and left ventricular mass index (LVMI). More importantly, we also assessed relevant outcomes in terms of serum indicators and functional capacities: B-type natriuretic peptide, amino-terminal peptide of procollagen type-III (PIIINP), QoL, and 6-min walking distance.

### Statistical analysis

For categorical variables, we calculated the relative risk (RR) and the absolute risk reduction (RD), as well as the corresponding 95% confidence intervals (CIs) for the outcome variables of interest, using the DerSimonian and Laird random effects model. Quantitative outcomes changing between baseline and follow-up were summarized and compared between the treatment and control groups using the weighted mean difference (WMD) and 95% CI, unless the outcomes used different scales, when the standardized mean difference (SMD) and 95% CI were used. The random-effects model using the DerSimonian and Laird method was used irrespective of heterogeneity because we anticipated heterogeneity between the trials [23]. *A priori*, we defined significant heterogeneity between trials as an I^2^ value of >50% [24]. We assessed the evidence of publication bias using a funnel plot with an Eggers test [25]. A two-sided *P*value <0.05 was considered statistically significant for all analyses.

Predefined subgroup analyses were conducted, *a priori*, according to the PEF subtypes (HF-PEF and MI-PEF), treatment durations (6 to 11 months, and ≥12 months), and MRA agent used (spironolactone, canrenoate, oreplerenone). If a given trial could be split into two or more separate studies, based on different treatment time points, the study with the longest follow-up was included in the meta-analyses. If a given trial could be split into two MRA groups with different doses, the group receiving the standard dose was included in the meta-analyses. Sensitivity analyses were conducted using sequential omission of a single study from the total studies and evaluating the influence of each study on the pooled effect estimates. All analyses were performed using Stata, version 11.2 (Stata Corp, College Station, TX, USA).

## Results

**Figure 1:**
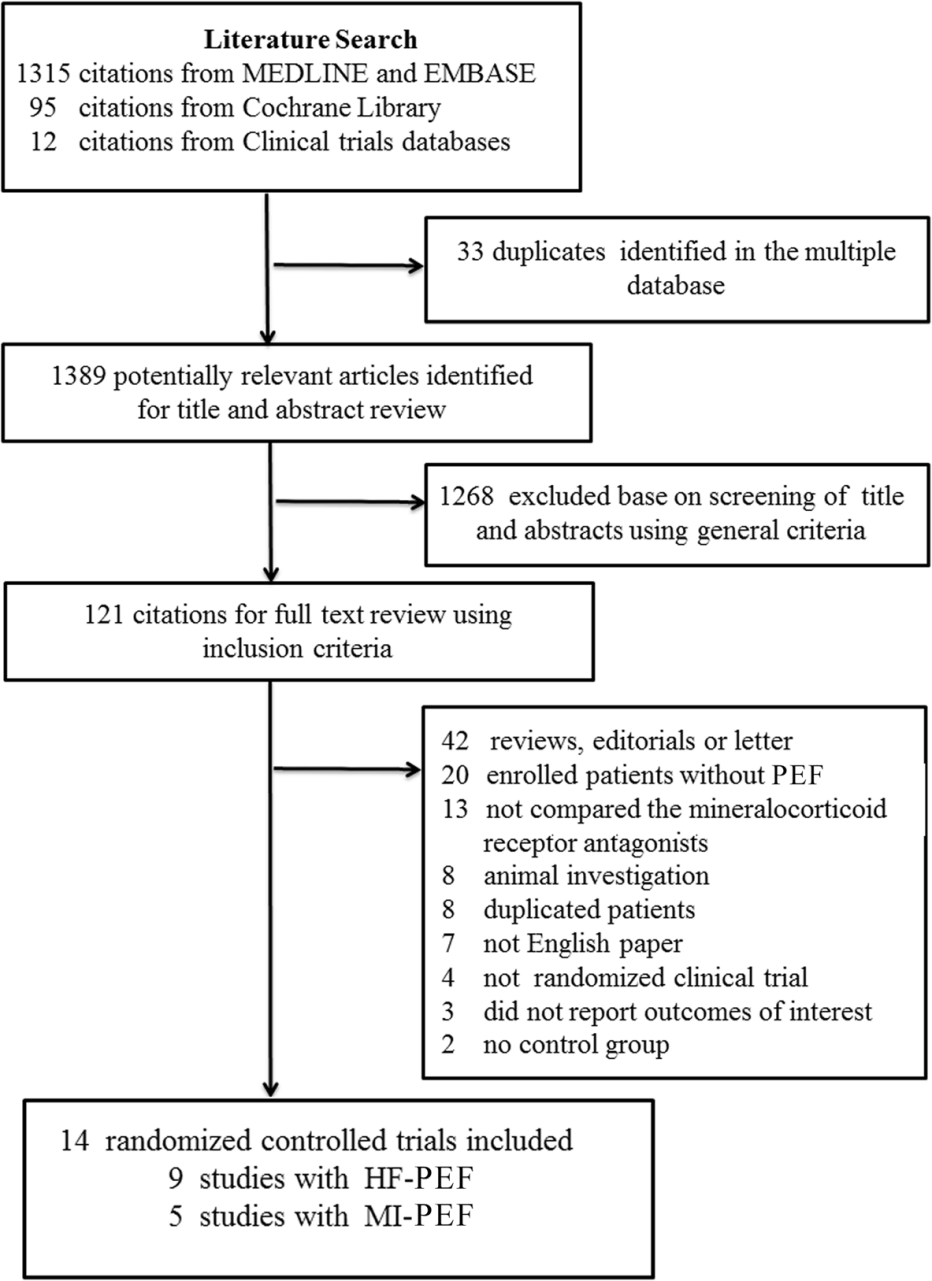
**Flowchart of study search and selection in this meta-analysis.** MI, Myocardial infarction; MRAs, Mineralocorticoid receptor antagonists; RCTs, Randomized controlled trials.

Figure 1 displays the study selection flow diagram. The primary search for randomized clinical trials on MRAs and PEF generated 1,422 potentially relevant articles. After screening the titles and abstracts of all studies, 121 full-text articles met the general inclusion criteria and were reviewed for strict inclusion or exclusion criteria. Finally, 14 RCTs were included [5,14-19,26-32].

### Study characteristics and quality

All trials (Table 1) were parallel arm trials, with a mean (± SD) duration of 11.75 ± 10.36 (range, 6 to39.6) months. Of the included trials, nine were conducted in HF-PEF (n = 4,127) [14,16,26-32] and five (n = 2,301) in MI-PEF patients [5,15,17-19]. Thus, a total of 6,428 participants were randomly assigned to receive MRAs (n = 3,249), placebo (n = 2,861), standard therapy (n = 301), or active comparator treatment (n = 17). The withdrawal rates were comparable between the MRA treatment groups (9.86%) and control groups (10.79%). Spironolactone was the predominant MRA used (eight trials) [5,14,16,19,27-29,32], followed by eplerenone (four trials) [15,17,30,31] and canrenoate (two trials) [18,26]. Placebo controls were used in all except four trials. Of these four trials, one used active comparators (ACE inhibitor [26]) and the other three used standard therapy [5,17,19]. Background medical therapy was inconsistently reported. The Treatment of Preserved Cardiac Function Heart Failure with an Aldosterone Antagonist (TOPCAT) trial [14] accounted for more than half of the patients in this meta-analysis. The Jadad scores of individual trials ranged from 1 to 5 (mean, 3.36 ± 1.34) and only three trials [16,17,32] had clear allocation concealment (Additional file 3: Table S1). Patient characteristics for the included studies are presented in Additional file 3: Tables S2 and S3.

**Table 1:** **Characteristics of included studies**

**Study (year) [Ref.]**	**Country**	**Patients**	**Inclusion criteria**	**Intervention: dose (mg/d)**	**Number randomized (withdrawals or dropouts)**	**Follow-up (months)**
Grand I (2002) [26]	USA	HF-PEF and HBP	LVEF >50%; BP >140/90 mmHg; LV diastolic dysfunction; normal renal function	Canrenone:50	17 (NR)	6
				ACEI	17 (NR)	
Mottram (2004) [27]	Australia	HF-PEF and HBP	LVEF >50%; NYHA II; E/A <1	Spironolactone:25	15 (NR)	6
				Placebo	15 (NR)	
Roongsritong (2005) [28]	USA	Elderly with HF-PEF	LVEF >45%; Age:60–85 years; mild diastolic dysfunction	Spironolactone:25	15 (1)	4
				Placebo	15 (1)	
Orea-Tejeda (2007) [29]	Mexico	HF-PEF	LVEF >40%; shortening fraction = 28%	Spironolactone:25–50	14 (NR)	13.79
				Standard therapy	14 (NR)	
Mak (2009) [30]	Ireland	HF-PEF	LVEF >45%; NYHA IV; BNP ≥100 pg/mL; diastolic dysfunction	Eplerenone:25	24 (0)	12
				Standard therapy	20 (2)	
RAAM-PEF trial (2011) [31]	USA	HF-PEF	LVEF >50%; NYHA II or III; BNP ≥100 pg/mL	Eplerenone:25 (titrated to 50)	23 (0)	7
				Placebo	23 (2)	
Aldo-DHF trial (2013) [16]	Germany and Austria	HFPEF	LVEF >50%; NYHA II or III; Diastolic dysfunction grade ≥ I	Spironolactone:25	213 (9)	11.6
				Placebo	209 (13)	
Kurrelmeyer (2014) [32]	USA	Elderly women with HF-PEF	LVEF >50%; NYHA II or III; E/e' > 15; BNP >62 pg/mL	Spironolactone:25	24 (0)	6
				Placebo	24 (0)	
TOPCAT trial (2014) [14]	Americas, Russia, and Georgia	HF-PEF	LVEF >45%; control blood pressure; BNP ≥100 pg/mL or NT-proBNP ≥360 pg/mL, diastolic dysfunction (grade ≥ I)	Spironolactone:15–45	1722 (160)	39.6
				Placebo	1723 (151)	
DiPasquale (2005) [18]	Italy	MI-PEF	LVEF >40%; ST >1 mm in the peripheral leads and/or >2 mm in precordial leads	Canrenoate:25	341 (33)	6
				placebo	346 (30)	
Kayrak (2010) [19]	Turkey	MI-PEF	LVEF >40%; successfully revascularized patients with AMI	Spironolactone:25	71 (16)	6
				Standard therapy	71 (16)	
Kampourides (2012) [17]	Greece	MI-PEF	LVEF >40%; AMI 1 day to 7 days	Eplerenone:25	210 (9)	30
				Standard therapy	140 (38)	
Vatankulu (2013) [5]	Turkey	MI-PEF	LVEF >40%; successfully revascularized patients with AMI	Spironolactone:25	54 (NR)	6
				Standard therapy	56 (NR)	
REMINDER trial (2014) [15]	European countries	MI-PEF	LVEF >40%; successfully revascularized patients with AMI	Eplerenone: 25 (titrated to 50)	506 (82)	10.5
				Placebo	506 (79)	

ACEI, Angiotensin-converting enzyme inhibitor; AMI, Myocardial infarction; BNP, B-type natriuretic peptide; E/A ratio, the ratio of early to late diastolic transmitral flow; E/e', an echocardiographic estimate of filling pressure for assessment of diastolic function; HBP, High blood pressure; HF-PEF, Heart failure with preserved systolic function; LVEF, Left ventricular ejection fraction; MI-PEF, Myocardial infarction with preserved systolic function; NR, Not reported; NT-proBNP, Amino terminal pro-B-type natriuretic peptide; NYHA, New York Heart Association functional class.

### Effect of MRAs on clinical outcomes

**Figure 2:**
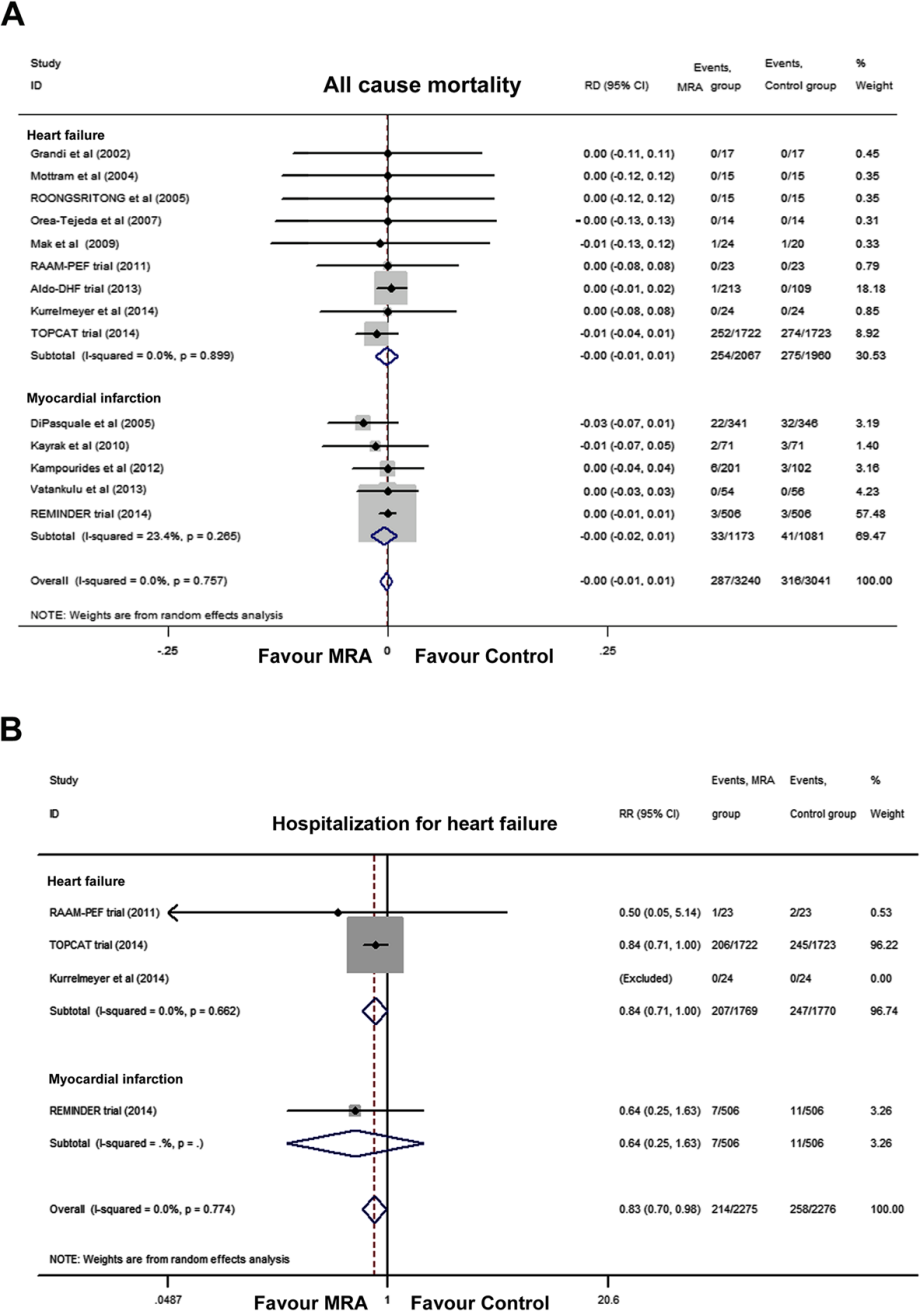
**Pooled analyses of all-cause mortality and hospitalization for heart failure in the mineralocorticoid receptor antagonist group versus controls. (A)** All-cause hospitalization; **(B)** Hospitalization due to heart failure. CI, Confidence interval; MRA, Mineralocorticoid receptor antagonist; RD, Absolute risk reduction; RR, Relative risk.

The combined data from the 14 RCTs did not show a significant association between MRA treatment and reduced all-cause mortality in PEF patients (RD, −0.00; 95% CI, −0.01 to 0.01; *P* = 0.71; I^2^ = 0%; Figure 2A, and RR, 0.90; 95% CI, 0.78 to 1.04; *P* = 0.17; I^2^ = 0%; Additional file 4: Figure S1A). Treatment with MRAs did not significantly reduce the incidence of all-cause mortality in the nine HF-PEF or in the five MI-PEF trials (*P* = 0.88 and *P* = 0.60, respectively). When analyzed by drug type, none of the individual therapies improved outcomes compared with the control group (spironolactone, *P* = 0.29; canrenoate, *P* = 0.18; eplerenone, *P* = 0.97; Additional file 4: Figure S1B). None of the individual studies significantly influenced the pooled all-cause mortality estimate in the leave-one-out sensitivity analysis (Additional file 4: Figure S1C); publication bias was not observed (*P* = 0.31, Additional file 4: Figure S1D).

Four studies [14,15,31,32] reported the outcomes of hospitalization due to HF involving 4,551 participants and 472 events. Overall, MRA treatment was associated with a reduced risk of hospitalization due to HF (RR, 0.83; 95% CI, 0.70 to 0.98; *P* = 0.03; I^2^ = 0%; Figure 2B, and RD, −0.01; 95% CI, −0.03 to 0.00; *P* = 0.05; I^2^ = 0%; Additional file 4: Figure S2A); a weight of 96.2% came from the TOPCAT trial. None of the individual studies influenced the pooled estimate of hospitalization due to HF (Additional file 4: Figure S2B); publication bias was not observed (*P* = 0.74; Additional file 4: Figure S2C).

Four studies reported the composite outcomes for deaths due to cardiovascular causes, aborted cardiac arrest, or hospitalization due to HF. Overall, MRA treatment did not significantly reduce the incidence of the composite outcome (RR, 0.89; 95% CI, 0.79 to 1.01; *P* = 0.07; I^2^ = 0%, Additional file 4: Figure S3A). When analyzed according to PEF subtype, a significant benefit was not observed for either HF-PEF (*P* = 0.18) or MI-PEF (*P* = 0.14) patients. Given the marked regional variation in event rates observed in the TOPCAT trial, we performed a separate pooled analysis, excluding patients randomized into TOPCAT trial from Russia and the republic of Georgia. The results showed that MRA treatment significantly reduced the incidence of composite outcome of death from cardiovascular causes, aborted cardiac arrests, or hospitalizations due to HF in PEF patients (RR, 0.85; 95% CI, 0.74 to 0.96; *P* = 0.01; I^2^ = 0%; Additional file 4: Figure S3B).

### Effect of MRAs on echo indexes of diastolic function

**Figure 3:**
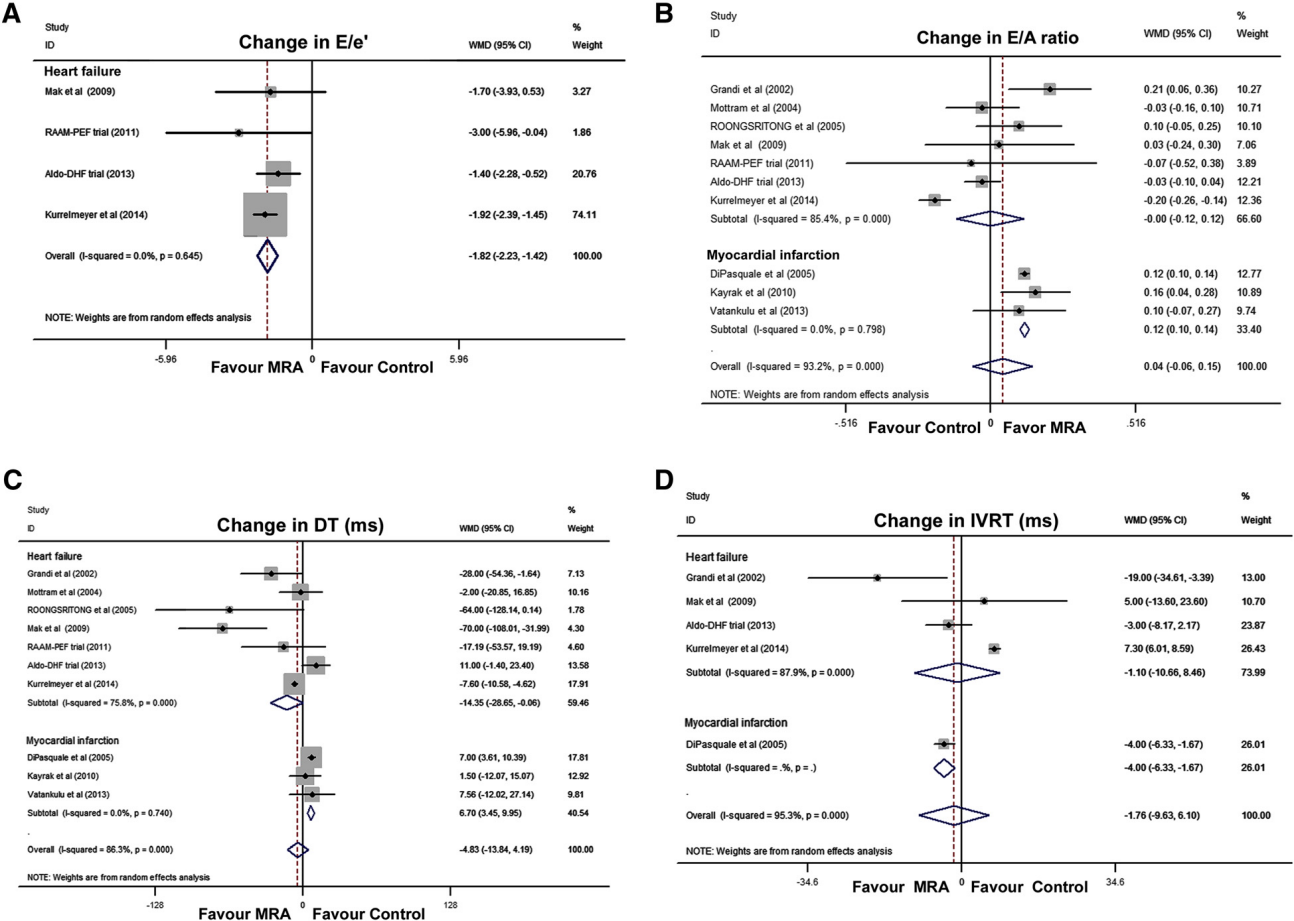
**Forest plots for effect of MRAs on echo indexes of diastolic function. (A)** Changes in E/e'; **(B)** E/A ratio changes; **(C)** DT changes; **(D)** IVRT changes. CI, Confidence interval; DT, E-wave deceleration time; E/A ratio, the ratio of early to late diastolic transmitral flow; E/e', an echocardiographic estimate of filling pressure for assessment of diastolic function; IVRT, Isovolumic relaxation time; MRA, Mineralocorticoid receptor antagonist; WMD, Weighted mean difference.

Overall, E/e', reported in 460 patients enrolled in four RCTs, was significantly improved following MRA treatment (WMD, −1.82; 95% CI, −2.23 to −1.42; I^2^ = 0%; Figure 3A), without evidence of publication bias (*P* = 0.36). None of the individual studies significantly influenced the pooled estimate for E/e' (Additional file 4: Figure S4A). The E/A ratio was the most common diastolic function variable, reported in 1,535 patients enrolled in 10 RCTs. Using data from three trials involving MI-PEF patients, MRA treatment significantly improved the E/A ratio (WMD, 0.12; 95% CI, 0.10 to 0.14; Figure 3B). However, the effect estimates for HF-PEF patients did not show improved E/A ratios (*P* = 0.97; Figure 3B). When analyzed by drug type, canrenoate treatment was associated with significant improvement in the E/A ratio (WMD, 0.13; 95% CI, 0.07 to 0.20; I^2^ = 29.8%; Additional file 4: Figure S4B). Treatment duration did not influence the pooled estimate (Additional file 4: Figure S4C).

For deceleration time, significance was attained among both HF-PEF (WMD, −14.35; 95% CI, −28.65 to 0.06; I^2^ = 75.8%; Figure 3C) and MI-PEF (WMD, 6.70; 95% CI, 3.45 to 9.95; I^2^ = 0%; Figure 3C) patients. Among the independent trials, overall estimates did not reach significance for IVRT (*P* = 0.66; Figure 3D).

### Effect of MRAs on indexes of cardiac structure and function

**Figure 4:**
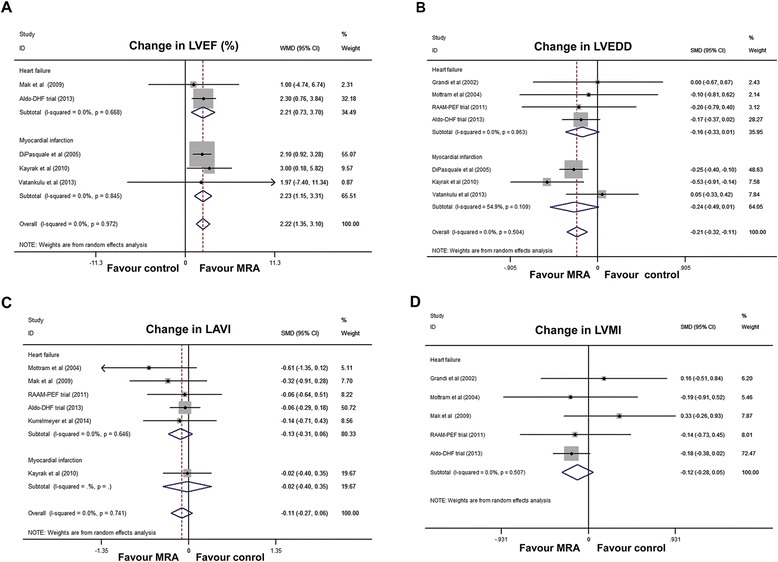
**Forest plots for effect of MRAs on indexes of cardiac structure and function. (A)** LVEF changes; **(B)** LVEDD changes; **(C)** LAVI changes; **(D)** LVMI changes. CI, Confidence interval; LAVI, Left atrial volume index; LVEDD, Left ventricular end-diastolic diameter; LVEF, Left ventricular ejection fraction; LVMI, Left ventricular mass index; SMD, Standardized mean difference; MRA, Mineralocorticoid receptor antagonist; WMD, Weighted mean difference.

With all trials included in the meta-analysis, improvement in LVEF (WMD, 2.22; 95% CI, 1.35 to 3.10; I^2^ = 0%; Figure 4A) and left ventricular end diastolic diameter (LVEDD) (SMD, −0.21; 95% CI, 0.32 to −0.11; I^2^ = 0%; Figure 4B) was apparent. No publication bias was evident (*P* = 0.94 and *P* = 0.61, respectively; Additional file 4: Figure S5A and S5B, respectively). Subgroup analyses, according to PEF subtype, found that the improvement in LVEF remained significant in both the HF-PEF and MI-PEF groups (Figure 4B). Subgroup analysis by treatment duration found that the MRA benefit on LVEDD was significant over 6 months, but not over 12 months (Additional file 4: Figure S5C).

For LAVI, the overall effect estimates did not demonstrate any significant benefit of MRA treatment (WMD, −0.11; 95% CI, −0.27 to 0.06; I^2^ = 0%; *P* = 0.26; Figure 4C). Similarly, MRA treatment was not associated with significant LVMI improvement (WMD, −0.12; 95% CI, −0.28 to 0.05; I^2^ = 0%; *P* = 0.22; Figure 4D). Further subgroup analyses failed to demonstrate significance for LAVI and LVMI (Figure 4D).

### Effect of MRAs on functional capacity and serum indicators

**Figure 5:**
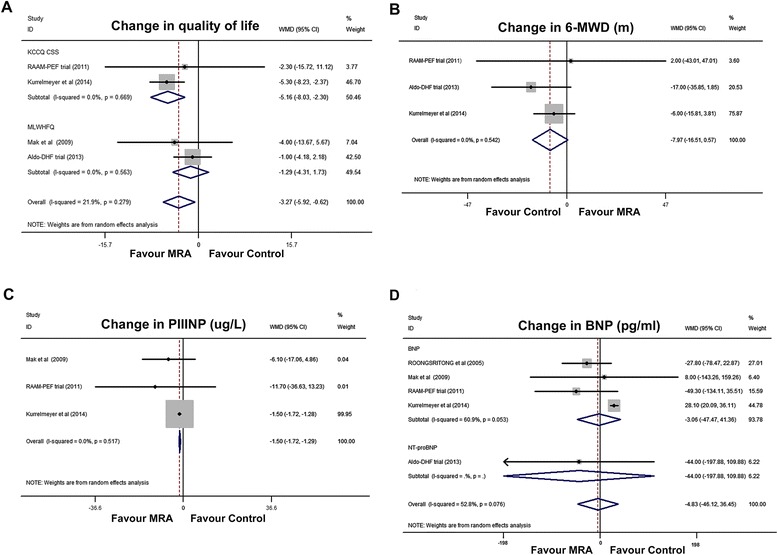
**Forest plots for effect of mineralocorticoid receptor antagonists on serum indicators and functional capacity. (A)** Quality of life changes; **(B)** 6-MWD changes; **(C)** PIIINP changes; **(D)** BNP changes. 6-MWD, 6-minute walk distance; BNP, B-type natriuretic peptide; CI, Confidence interval; KCCQ CSS, Kansas City Cardiomyopathy Questionnaire clinical summary score; PIIINP, Serum amino-terminal peptide of procollagen type-III; MLWHF, Minnesota Living with Heart Failure questionnaire; MRA, Mineralocorticoid receptor antagonist; WMD, Weighted mean difference.

The pooled analyses of QoL and 6-min walk distance are shown in Figure 5A and B, respectively. When the QoL was measured using the Kansas City Cardiomyopathy Questionnaire clinical summary score (KCCQ CSS), MRA treatment was associated with a significant improvement in QoL in HF-PEF patients (WMD, −5.16; 95% CI, −8.03 to −2.30; I^2^ = 0%; *P* < 0.0001; Figure 5A). However, the Minnesota Living with Heart Failure questionnaire QoL measurements did not improve significantly. In the three HF-PEF trials that reported 6-min walk distance, a non-significant change was seen (WMD, −7.97; 95% CI, −16.51 to 0.57; I^2^ = 0%; *P* = 0.07; Figure 5B).

Pooling the results of all qualified trials found a significant serum PIIINP level reduction (WMD, −1.50; 95% CI, −1.72 to −1.29; I^2^ = 0%; Figure 5C), with significant publication bias (*P* = 0.01). As for B-type natriuretic peptide (or amino terminal-proB-type natriuretic peptide), the pooled analysis did not show a significant reduction in the MRA group (WMD, −4.83; 95% CI, −46.12 to 36.45; I^2^ = 52.8%; *P* = 0.82; Figure 5D).

### Safety and adverse events

Hyperkalemia was reported in all but one trial [28]. Over a mean follow-up period of 12.19 months, the MRA group showed a higher rate of hyperkalemia (>5.5 mmol/L) with 12.15% of the MRA groups and 6.16% of the control groups reporting hyperkalemia (*P* < 0.001). The TOPCAT trial, which had the highest reported rates of hyperkalemia, involved spironolactone treatment (15 to 45 mg). Renal failure, using definitions from within each trial, occurred in 1.91% of MRA patients and in 0.37% of control patients. Gynecomastia was reported in seven studies [5,14,16,19,26,29,31] involving MRA (2.81%) and control (0.30%) patients.

## Discussion

In this meta-analysis of RCTs involving 6,248 patients, the effects of MRAs on patients with either MI-PEF or HF-PEF were evaluated. MRA treatment reduced the risk of hospitalization due to HF, improved QoL, reduced the E/e' or E/A ratio, increased LVEF, and reduced LVEDD and PIIINP levels in PEF patients. However, significant all-cause mortality benefits were not seen.

As patients with HF-PEF are usually older than HF-REF patients, hospitalization due to HF is increasing and represents a major burden in these patients [1,33], and emphasizes the growing need for effective, evidence-based therapies. However, previous pharmacological interventions, such as angiotensin-converting enzyme inhibitors [34], angiotensin receptor blockers [35], and beta-blockers [36], have failed to show a significant reduction in hospitalizations due to HF. This meta-analysis provides important insights into the potential efficacy of MRA treatment for reducing the rate of hospitalizations due to HF in PEF patients, without increasing mortality. Reducing hospitalizations due to HF may help lower hospitalization costs and improve patient QoL. Additionally, significant MRA treatment benefits on composite outcome of death from cardiovascular causes, aborted cardiac arrests, or hospitalizations were observed after excluding patients recruited from Russia and the Republic of Georgia into the TOPCAT trial. HF-PEF patients from these jurisdictions, in that trial [14], had extremely low placebo event rates, incompatible with those in prior HF-PEF studies [35,37]. The separate meta-analysis, excluding this population, might provide a more realistic insight into the effectiveness of MRAs in HF-PEF patients. Furthermore, we demonstrated that MRA treatment was associated with a significant improvement in QoL, measured by the KCCQ CCS. The KCCQ CCS has been reported to be a valid and reliable measure of health status and QoL in HF-PEF patients [38]. Since the HF-PEF patients were elderly and typically demonstrated multiple comorbidities that might affect their mobility, it is not surprising that MRA treatment improved KCCQ CCS scores in these patients, but not exercise tolerance [39]. Therefore, MRA treatment could be an option in PEF patients to improve their QoL.

Another encouraging finding from this meta-analysis was that MRAs improved both diastolic and systolic functions in PEF patients. Left ventricular diastolic dysfunction is the major underlying cardiac pathophysiology of PEF patients, and worse diastolic dysfunction has been associated with an increased risk of mortality [40]. However, earlier pharmacotherapy did not achieve a significant improvement in diastolic function in HF-PEF patients [7,41,42]. The present meta-analysis supports the potential clinical value of MRAs for improving diastolic function in PEF patients. We also found that MRA treatment significantly reduced the E/e' in HF-PEF patients and the E/A ratio in MI-PEF patients. Interestingly, MRAs were not associated with a significant reduction in the E/A ratio in HF-PEF patients. This may be because this ratio is rather complicated and cannot provide unequivocal evidence of diastolic dysfunction in HF-PEF [43], whereas E/e' was demonstrated to be the most accurate, non-invasive index of diastolic function in HF-PEF [43]. Our meta-analysis also found that MRA administration led to increased LVEF in PEF patients. A previous meta-analysis demonstrated that MRAs improved systolic function in HF-REF [44]. Thus, the present meta-analysis shows that the beneficial effect of MRAs on systolic function also extends to PEF patients.

Although the favorable impact of MRAs on cardiac remodeling is well known in patients with HF and reduced LVEF [44], the effect of MRAs in patients with preserved systolic function remained uncertain. This meta-analysis demonstrated that MRA administration could reverse cardiac remodeling in patients with preserved systolic function through a reduction of LVEDD and PIIINP levels. A subgroup analysis of LVEDD, based on treatment duration, found that the reduction became insignificant as the duration increased. This finding is consistent with a previous meta-analysis focusing on the effect of MRAs on cardiac structure in patients with left ventricular dysfunction [44]. As PIIINP level has been proposed as an indicator of cardiac remodeling and poor clinical prognosis [45], a reduction in serum PIIINP level might reflect the beneficial effects of MRAs on cardiac remodeling.

The benefits of MRAs on PEF patients are mainly attributed to the improvement of endothelial function and cardiac remodeling, as well as the decrease of myocardial fibrosis. Experimental evidence indicates that aldosterone-induced mineralocorticoid receptor activation provides an important unifying mechanism for many of the pathologic alterations of HF-PEF and MI-PEF [46,47]. The MRAs, through direct inhibition of aldosterone, were demonstrated to reduce myocardial fibrosis, improve vascular compliance and endothelial function, decrease inflammation and oxidative stress, and reduce the release of norepinephrine [48]. These changes likely account for the diastolic function improvements seen on echocardiography and the reduction in collagen markers such as serum PIIINP level. MRA treatment was also associated with an increased risk of hyperkalemia and elevated serum creatinine levels. Our findings underscore the importance of monitoring electrolyte disorders and serum markers of kidney function during MRA treatment in clinical practice.

Several issues should be considered in the interpretation of our results. First, this meta-analysis was limited by the discrepancies in PEF diagnostic criteria employed in the clinical trials. The diagnosis of HF-PEF is still challenging because various criteria have been proposed to define patients with “diastolic HF” [49]. The included RCTs had differing ejection fraction cut-off criteria (range, 40 to 50%) and challenges in diagnostic criteria for PEF, and may have resulted in a heterogeneous population. Nevertheless, patients with an ejection fraction of 40 to 50%, defined as borderline and intermediate PEF in the American College of Cardiology Foundation/American Heart Association guidelines [49], were characteristically and prognostically similar to those with an ejection fraction ≥ 50% [50]. Therefore, our meta-analysis does suggest a potential MRA treatment benefit for PEF patients. Second, some publication bias might exist in this meta-analysis, as we only included articles published in English. However, our statistical tests reported a low probability of publication bias in the pooling analysis. Third, different follow-up durations in the included trials might have produced heterogeneity, which limited the interpretation of pooled effect estimates. Finally, as the reported totals for all-cause mortality and hospitalizations due to HF were low, the assessment of the effect on clinical outcomes in PEF patients was of limited power. Despite the majority of evidence regarding clinical outcomes coming from the recently reported TOPCAT trial [14], the findings of previous trials appear consistent.

## Conclusions

This meta-analysis of RCTs in PEF patients demonstrated that MRA treatment may exert beneficial effects, including reduced hospitalizations due to HF, improved QoL and diastolic function, and cardiac remodeling reversal, without an effect on all-cause mortality. The significant increase in hyperkalemia and serum creatinine level associated with MRA treatment underscores the need for careful monitoring of electrolyte disorders and serum markers of kidney function in clinical practice. Further large-scale RCTs are needed to confirm the clinical indications for this medication class.

## Additional files

Additional file 1:
**PRISMA checklist.**
Additional file 2:
**Search Strategies.**
Additional file 3: Table S1. Quality Assessment of included RCTs in this meta-analysis. **Table S2.** Characteristics of patients in the included studies. **Table S3.** Characteristics of patients in the included studies. Additional file 4: Figure S1. Effect of MRAs on all-cause mortality. (A) Pooled effect estimate RR on all-cause mortality; (B) Subgroup analysis by drug type; (C) Leave-one-out analysis; (D) Funnel plots for all-cause mortality. CI, Confidence interval; MRA, Mineralocorticoid receptor antagonist; RR, Relative risk; SE, Standard error. **Figure S2.** Effect of MRAs on hospitalization for heart failure. (A) Pooled effect estimate RD on hospitalization for heart failure; (B) Leave-one-out analysis; (C)Funnel plots for hospitalization for heart failure. CI, Confidence interval; MRA, Mineralocorticoid receptor antagonist; RD, Absolute risk reduction; SE, Standard error. **Figure S3.** Effect of MRAs on composite outcomes for deaths due to cardiovascular causes, aborted cardiac arrest, or hospitalization due to heart failure. (A) Pooled effect estimate RR on composite outcomes; (B) Pooled analysis excluding patients randomized into TOPCAT trial from Russia and the Republic of Georgia on composite outcomes.CI, Confidence interval; MRA, Mineralocorticoid receptor antagonist; RR, Relative risk; SE, Standard error. **Figure S4.** Funnel plots and subgroup analyses of echo indexes of diastolic function. (A) Leave-one-out analysis for E/e'; (B) Subgroup analyses by drug type; (C) Subgroup analyses by treatment duration. CI, Confidence interval; E/A ratio, the ratio of early to late diastolic transmitral flow; E/e', an echocardiographic estimate of filling pressure for assessment of diastolic function; MRA, Mineralocorticoid receptor antagonist; WMD, Weighted mean difference. **Figure S5.** Subgroup analyses of indexes of cardiac structure and function. (A) Funnel plots for LVEF; (B) Subgroup analyses by LVEDD; (C) Subgroup analyses by treatment duration. LVEDD, Left ventricular end-diastolic diameter; LVEF, Left ventricular ejection fraction; LVMI, Left ventricular mass index; SMD, Standardized mean difference; MRA, Mineralocorticoid receptor antagonist; WMD, Weighted mean difference.

## Abbreviations

CIs: Confidence intervals
E/A: Ratio of early to late diastolic transmitral flow
E/e': Estimate of filling pressure used to assess diastolic function
HF: Heart failure
HF-PEF: HF with preserved ejection fraction
HF-REF: HF patients with a reduced ejection fraction
IVRT: Isovolumic relaxation time
KCCQ CSS: Kansas City Cardiomyopathy Questionnaire clinical summary score
LAVI: Left atrial volume index
LVEDD: Left ventricular end diastolic diameter
LVEFs: Left ventricular ejection fractions
LVMI: Left ventricular mass index
MI: Myocardial infarction
MI-PEF: PEF after MI
MRAs: Mineralocorticoid receptor antagonists
PEF: Preserved ejection fraction
PIIINP: Peptide of procollagen type-III
QoL: Quality of life
RCTs: Randomized controlled trials
RD: Risk reduction
RR: Relative risk
SMD: Standardized mean difference
TOPCAT: Treatment of Preserved Cardiac Function Heart Failure with an Aldosterone Antagonist
WMD: Weighted mean difference
