# Supplementary material for: Effects of mineralocorticoid receptor antagonists in patients with preserved ejection fraction: a meta-analysis of randomized clinical trials
Source: BMC Med. 2015 Jan 19;13:10. doi: 10.1186/s12916-014-0261-8 (PMC4307751; doi:10.1186/s12916-014-0261-8)
Supplement: Additional file 2: — Search Strategies. [file 12916_2014_261_MOESM2_ESM.docx]

**Additional file 2**

Search strategies for EMBASE (30 June 2014)

**#1** 'mineralocorticoid antagonist'/exp OR 'mineralocorticoid antagonist'

**#2** 'aldosterone antagonist'/exp OR 'aldosterone antagonist'

**#3** 'aldosterone blockade':ab,ti

**#4** 'aldosterone receptor antagonist':ab,ti

**#5** 'spironolactone'/exp OR 'spironolactone'

**#6** 'aldactone' OR 'aldactone'/exp OR aldactone

**#7** 'inspra'/exp OR 'inspra'

**#8** 'canrenoic acid'/exp OR 'canrenoic acid'

**#9** 'canrenoate potassium'/exp OR 'canrenoate potassium'

**#10** 'canrenone'/exp OR 'canrenone':ab,ti

**#11** 'aldactone'/exp OR 'aldactone':ab,ti

**#12** ' eplerenone '/exp OR ' eplerenone ':ab,ti

**#13** #1 OR #2 OR #3 OR #4 OR #5 OR #6 OR #7 OR #8 OR #9 OR #10 OR #11 OR #12

**#14**  'myocardial infarction'/exp OR 'myocardial infarction'

**#15** 'diastolic heart failure'/exp OR 'diastolic heart failure'

**#16** 'diastole':ab,ti

**#17** 'diastolic dysfunction':ab,ti

**#18**  'heart failure with preserved ejection fraction':ab,ti

**#19** 'heart failure preserved ejection fraction':ab,ti

**#20** 'preserved ejection fraction':ab,ti

**#21**  'left ventricular diastolic failure':ab,ti

**#22**  'preserved left ventricular ejection fraction':ab,ti

**#23** 'heart failure with normal ejection fraction':ab,ti

**#24** 'preserved left ventricular function':ab,ti

**#25**  #14 OR #15 OR #16 OR #17 OR #18 OR #19 OR #20 OR #21 OR #22 OR #23 OR #24

**#26** 'randomized controlled trial'/exp OR 'randomized controlled trial'

**#27**  'clinical trial'/exp OR 'clinical trial'

**#28**  'random$':ab,ti

**#29** 'placebo$':ab,ti

**#30**  'double blind method':ab,ti

**#31** 'single blind method'/exp OR 'single blind method'

**#32** 'triple blind method'/exp OR 'triple blind method'

**#33** 'double blind clinical study':ab,ti

**#34** 'single blind mask$':ab,ti

**#35** 'crossover study':ab,ti

**#36** #26 OR #27 OR #28 OR #29 OR #30 OR #31 OR #32 OR #33 OR #34 OR #35

**#37** #13 AND #25 AND #36

**#38** #37 AND [english]/lim AND [humans]/lim AND [2000-2015]/py

Search strategies for Medline (30 June 2014)

**#1** aldosterone antagonists[MeSH Terms]

**#2** aldosterone blocks[Title/Abstract]

**#3** aldosterone receptor antagonist[Title/Abstract]

**#4** aldosterone blockade[Title/Abstract]

**#5** spironolactone[MeSH Terms]

**#6** spironolactone[Title/Abstract]

**#7** aldactone[Title/Abstract]

**#8** eplerenone[Title/Abstract]

**#9** inspra[Title/Abstract]

**#10** canrenoic acid[Title/Abstract]

**#11** canrenoate potassium[MeSH Terms]

**#12** (canrenone[MeSH Terms]) OR canrenone[Title/Abstract]

**#13** (aldactone[MeSH Terms] OR aldactone[Title/Abstract])

**#14** (mineralocorticoid receptor[MeSH Terms]) OR mineralocorticoid receptor[Title/Abstract]

**#15** #1 OR #2 OR #3 OR #4 OR #5 OR #6 OR #7 OR #8 OR #9 OR #10 OR #11 OR #12 OR #13 OR #14

**#16**  heart failure, diastolic[MeSH Terms]

**#17** diastolic heart failure[Title/Abstract]

**#18** heart failure with normal ejection fraction[Title/Abstract]

**#19** heart failure with preserved ejection fraction[Title/Abstract]

**#20**  left ventricular diastolic failure[Title/Abstract]

**#21** myocardial infarction[MeSH Terms]

**#22** acute myocardial infarction[Title/Abstract]

**#23** #16 OR #17 OR #18 OR #19 OR #20 OR #21 OR #22

**#24** randomized controlled trial[MeSH Terms]

**#25**  clinical trial[Publication Type]

**#26** random$[Title/Abstract]

**#27** randomized[Title/Abstract]

**#28**  (placebo[Title/Abstract]) OR placebos[Title/Abstract]

**#29**  ((double blind method[Text Word]) OR single blind method[Text Word]) OR triple blind method[Text Word]

**#30** ((((double blind mask) OR double blind masks) OR single blind mask) OR triple blind mask) OR triple blind masks

**#31**  (crossover study[Text Word]) OR cross-over study[Text Word]

**#32** #24 OR #25 OR #26 OR #27 OR #28 OR #29 OR #30 OR#31

**#33**  #15 AND #23 AND #32

**#34** #33 Filters: Publication date from 2000/01/01 to 2014/06/30; Humans; English
