# Supplementary material for: Effects of mineralocorticoid receptor antagonists in patients with preserved ejection fraction: a meta-analysis of randomized clinical trials
Source: BMC Med. 2015 Jan 19;13:10. doi: 10.1186/s12916-014-0261-8 (PMC4307751; doi:10.1186/s12916-014-0261-8)
Supplement: Additional file 3: Table S1. — Quality Assessment of included RCTs in this meta-analysis. Table S2. Characteristics of patients in the included studies. Table S3. Characteristics of patients in the included studies. [file 12916_2014_261_MOESM3_ESM.docx]

**Additional file 3: Tables**

**Table S1:** **Quality assessment of included RCTs in this meta-analysis**

| **Study(year)** | **Randomization** | **Blinding** | **Drop-outs** | **Jadad score** | **clear allocation**  **concealment** |
| --- | --- | --- | --- | --- | --- |
| Grandi (2002)[26] | 1 | 0 | 0 | 1 | NO |
| Mottram (2004)[27] | 1 | 2 | 0 | 3 | NO |
| ROONGSRITONG(2005)[28] | 1 | 2 | 1 | 4 | NO |
| Orea-Tejeda (2007)[29] | 1 | 0 | 0 | 1 | NO |
| Mak (2009)[30] | 1 | 1 | 1 | 3 | NO |
| RAAM-PEF trial (2011)[31] | 1 | 2 | 1 | 4 | NO |
| Aldo-DHF trial (2013)[16] | 2 | 2 | 1 | 5 | NO |
| Kurrelmeyer (2014)[32] | 1 | 2 | 1 | 4 | Yes |
| TOPCAT trial (2014)[14] | 2 | 2 | 1 | 5 | Yes |
| DiPasquale (2005)[18] | 1 | 2 | 1 | 4 | NO |
| Kayrak (2010)[19] | 2 | 0 | 1 | 3 | NO |
| Kampourides (2012)[17] | 1 | 1 | 1 | 3 | Yes |
| Vatankulu (2013)[5] | 1 | 1 | 0 | 2 | NO |
| REMINDER trial (2014)[15] | 2 | 2 | 1 | 5 | NO |

**The Jadad scoring system for randomized controlled trials** (Jadad AR, Moore RA, Carroll D, Jenkinson C, Reynolds DJ, Gavaghan DJ, McQuay HJ: Assessing the quality of reports of randomized clinical trials: is blinding necessary? Controlled clinical trials 1996, 17:1-12.)

1. Was the study described as randomized (this includes the use of words such as randomly, random, and randomization)?

2. Was the study described as double blind?

3. Was there a description of withdrawals and dropouts?

**Scoring the items:** Either give a score of 1 point for each “yes” or 0 points for each “no”. There are no in-between marks.

**Give 1 additional 1 point if:**  For question 1 (Randomization), the method to generate the sequence of randomization was described and it was appropriate (table of random numbers, computer generated, etc.).

**and/or:**  If for question 2 (Blinding), the method of double blinding was described and it was appropriate (identical placebo, active placebo, dummy, etc.).

**Deduct 1 point if:** For question 1(Randomization), the method to generate the sequence of randomization was described and it was inappropriate (patients were allocated alternately, or according to date of birth, hospital number, etc.).

**and/or:**  For question 2(Blinding), the study was described as double blind but the method of blinding was inappropriate (e.g., comparison of tablet vs. injection with no double dummy).

**Table S2. Characteristics of patients in the included studies.**

| **Study** | **Country** | **Age**  **(year)** | **Female (%)** | **Mean**  **EF (%)** | **SBP**  **(mmHg)** | **DBP (mmHg)** | **K+**  **(mmol/L)** | **Creatinine (mg/dL)** | **BNP**  **(pg/mL)** |
| --- | --- | --- | --- | --- | --- | --- | --- | --- | --- |
| **Grand I (2002) [26]** |  |  |  |  |  |  |  |  |  |
| MRA group | USA | NR | NR | NR | 128 (7) | 79 (5) | 4.2 (0.2) | 0.92 (0.04) | NR |
| Control group |  | NR | NR | NR | 127 (7) | 76 (7) | 4.2 (0.1) | 0.92 (0.03) | NR |
| **Mottram (2004)[27]** |  |  |  |  |  |  |  |  |  |
| MRA group | Australia | 61 (6) | 60 | 68 (5) | 135 (17) | 80 (8) | 4.0 (0.4) | 0.79 (0.11) | 29.3 (26.8) |
| Control group |  | 62 (5) | 66 | 67 (4) | 130 (16) | 82 (8) | 3.9 (0.3) | 0.79 (0.11) | 29.7 (27.8) |
| **ROONGSRITONG (2005)[28]** |  |  |  |  |  |  |  |  |  |
| MRA group | USA | 71.0 (5.5) | 78 | 64.2 (4.9) | 144 (22) | 75 (14) | NR | NR | 54.9 (64.2) |
| Control group |  | 72.1 (6.9) | 78 | 67.4 (3.3) | 142 (21) | 72 (11) | NR | NR | 51.1 (52.7) |
| **Orea-Tejeda (2007)[29]** | Mexico |  |  |  |  |  |  |  |  |
| MRA group |  | 63.71 (21.61) | 71.4 | 48.79 (4.65) | 112 (12) | NR | NR | NR | NR |
| Control group |  | 64.79 (11.89) | 28.6 | 51.57 (11.71) | 114 (8) | NR | NR | NR | NR |
| **Mak (2009)[30]** |  |  |  |  |  |  |  |  |  |
| MRA group | Ireland | 80 (7.7) | 9 (38) | 63 (9.0) | 140 (20) | 80 (17) | 4.2 (0.4) | 1.3 (0.26) | 219 (157-317) |
| Control group |  | 79 (7.9) | 11 (55) | 64 (9.6) | 146 (20) | 79 (18) | 4.2 (0.5) | 1.3 (0.3) | 192 (132-330) |
| **RAAM-PEF trial (2011)[31]** |  |  |  |  |  |  |  |  |  |
| MRA group | USA | 72.2 (9.8) | 4.8 | 62.1 (5.0) | 129.7 (12.4) | 71.1 (11.0) | 4.1 (0.7) | 1.62 (0.50) | 254.9 (163.0) |
| Control group |  | 68.7 (9.1) | 8.7 | 62.5 (7.5) | 130.6 (10.7) | 67.9 (9.6) | 4.0 (0.52) | 1.43 (0.51) | 283.5 (211.6) |
| **Aldo-DHF trial (2013)[16]** |  |  |  |  |  |  |  |  |  |
| MRA group | Germany and  Austria | 67 (8) | 52 | 67 (8) | 135 (18) | 79 (10) | 4.2 (0.4) | NR | 179 (81-276) |
| Control group |  | 67 (8) | 53 | 68 (7) | 135 (18) | 80 (12) | 4.2 (0.4) | NR | 148 (80-276) |
| **TOPCAT trial (2014)[14]** |  |  |  |  |  |  |  |  |  |
| MRA group | Americas, Russia and Georgia | 68.7 (61-76) | 51.6 | 56 (51-61） | 130 (120-139) | 80 (70-80) | 4.3 (4.0-4.6) | 1.0 (0.9-1.2) | 236 (149-414) |
| Control group |  | 68.7 (61-76) | 51.5 | 56 (51-62） | 130 (120-140) | 80 (70-80) | 4.3 (4.0-4.6) | 1.1 (0.9-1.2) | 235 (141-410) |
| **Kurrelmeyer (2014)[32]** |  |  |  |  |  |  |  |  |  |
| MRA group | USA | 66.3 (2.2) | 100 | 62.5 (1.2) | 137.0 (4.1) | 72.4 (2.6) | NR | NR | NR |
| Control group |  | 76.4 (1.6) | 100 | 62.9 (1.2) | 133.1 (2.8) | 67.3 (1.6) | NR | NR | NR |
| **DiPasquale (2005)[18]** | Italy |  |  |  |  |  |  |  |  |
| MRA group |  | 62.6 (6) | 28.7 | 44.5 (6) | NR | NR | 3.6 (0.2) | 1.07 (0.12) | NR |
| Control group |  | 62.8 (5) | 29.5 | 44.7 (9) | NR | NR | 3.5 (0.4) | 1.06 (0.13) | NR |
| **Kayrak (2010)[19]** |  |  |  |  |  |  |  |  |  |
| MRA group | Turkey | 55.3 (10) | 81.8 | 50.5 (8.3) | 105.2 (9.7) | 67.0 (5.2) | 4.1 (0.4) | 0.99 (0.2) | NR |
| Control group |  | 57.2 (11.1) | 74.5 | 49.5 (8.0) | 105.0 (10.6) | 67.1 (6.8) | 4.2 (0.5) | 0.97 (0.29) | NR |
| **Kampourides (2012)[17]** | Greece |  |  |  |  |  |  |  |  |
| Total patents |  | 58 (50-68) | 17.8 | 57 (53-67) | 150 (130-160) | NR | 4.3 (4.1-4.6) | 1 (0.9-1.1) | 322 (95-679)* |
| **Vatankulu(2013)[5]** |  |  |  |  |  |  |  |  |  |
| MRA group | Turkey | 58 (9) | 15 | 49.1 (40-55) | NR | NR | NR | NR | NR |
| Control group |  | 57 (11) | 20 | 50.1 (41-55) | NR | NR | NR | NR | NR |
| **REMINDER trial (2014)[15]** |  |  |  |  |  |  |  |  |  |
| MRA group | European countries | 58.5 (10.8) | 17 | NR | 125.7 (18.7) | 76.0 (12.5) | 4.07 (0.46) | 0.91 (0.20) | NR |
| Control group |  | 57.8 (11) | 20.4 | NR | 126.7 (17.0) | 76.8 (11.5) | 4.04 (0.45) | 0.91 (0.21) | NR |

BNP, brain natriuretic peptide; DBP, diastolic blood pressure; EF, ejection fraction; SBP, systolic blood pressure; MRA, mineralocorticoid receptor antagonist; NR, not reported.

| **Study** | **Ischaemia (%)** | **Hypertension (%)** | **DM (%)** | **AF (%)** | **ACEI (%)** | **β-Blockers (%)** | **Diuretics (%)** | **CCB (%)** |
| --- | --- | --- | --- | --- | --- | --- | --- | --- |
| **Grandi[26]** |  |  |  |  |  |  |  |  |
| MRA group | NR | 100 | NR | NR | NR | NR | NR | NR |
| Control group | NR | 100 | NR | NR | NR | NR | NR | NR |
| **Mottram [27]** |  |  |  |  |  |  |  |  |
| MRA group | NR | 100 | 6.7 | NR | NR | 40 | 40 | 53 |
| Control group | NR | 100 | 0 | NR | NR | 20 | 27 | 60 |
| **ROONGSRITONG[28]** |  |  |  |  |  |  |  |  |
| MRA group | 42 | 85 | NR | NR | 54 | NR | NR | NR |
| Control group | 38 | 78 | NR | NR | 47 | NR | NR | NR |
| **Orea-Tejeda[29]** |  |  |  |  |  |  |  |  |
| MRA group | 42.9 | 85.7 | 28.6 | NR | 38.5 | 79.5 | 76.9 | 5.1 |
| Control group | 57.1 | 92.9 | 64.3 | NR | 29 | 79.7 | 62.3 | 13 |
| **Mak[30]** |  |  |  |  |  |  |  |  |
| MRA group | NR | 92 | 21 | 58 | 67 | 62 | 88 | 17 |
| Control group | NR | 90 | 35 | 60 | 60 | 75 | 90 | 25 |
| **RAAM-PEF trial[31]** |  |  |  |  |  |  |  |  |
| MRA group | 66.7 | 100 | 61.9 | 14.3 | 95.2 | 76.2 | 95.2 | 52.4 |
| Control group | 47.8 | 100 | 60.9 | 13 | 100 | 82.6 | 100 | 47.8 |
| **Aldo-DHF trial[16]** |  |  |  |  |  |  |  |  |
| MRA group | 43 | 92 | 17 | 6 | 78 | 69 | 55 | 22 |
| Control group | 37 | 91 | 16 | 4 | 76 | 75 | 52 | 28 |
| **TOPCAT trial[14]** |  |  |  |  |  |  |  |  |
| MRA group | 57.4 | 91 | 32.8 | 35.5 | 84.3 | 78.2 | 81.4 | 36.3 |
| Control group | 60.1 | 91.9 | 32.2 | 35.1 | 84.2 | 77.3 | 82.3 | 38.9 |
| **Kurrelmeyer[32]** |  |  |  |  |  |  |  |  |
| MRA group | 37.5 | 87.5 | 50 | 25 | 79.8 | 62.5 | 83.3 | 25 |
| Control group | 33.3 | 79.2 | 25 | 25 | 66.7 | 62.5 | 75 | 29.2 |
| **DiPasqual[18]** |  |  |  |  |  |  |  |  |
| MRA group | 100 | 35.8 | 38.7 | NR | 100 | 36.95 | NR | NR |
| Control group | 100 | 35 | 40.8 | NR | 100 | 3.97 | NR | NR |
| **Kayra [19]** |  |  |  |  |  |  |  |  |
| MRA group | 100 | 25.4 | 20 | NR | 90.9 | 98.1 | NR | NR |
| Control group | 100 | 29 | 18 | NR | 92.7 | 94.5 | NR | NR |
| **Kampouride [17]** |  |  |  |  |  |  |  |  |
| Total patents | 100 | 58 | 20 | 3 | 82.2 | 84 | 9 | 10 |
| **AkifVatankulu[5]** |  |  |  |  |  |  |  |  |
| MRA group | 100 | 28 | 17 | NR | 93 | 96 | NR | NR |
| Control group | 100 | 27 | 20 | NR | 82 | 51 | NR | NR |
| **REMINDER trial[15]** |  |  |  |  |  |  |  |  |
| MRA group | 100 | 47.6 | 12.8 | 1.6 | 82.6 | 87.9 | 10.7 | NR |
| Control group | 100 | 51.4 | 15.4 | 1.8 | 83.2 | 88.3 | 10.1 | NR |

**Table S3. Characteristics of patients in the included studies**

ACEI, angiotensin-converting enzyme inhibitor; AF, atrial fibrillation; CCB, calcium channel blockers; DM, diabetes mellitus; EF, ejection fraction; MRA, mineralocorticoid receptor antagonist; NR, not reported.
