# Supplementary figures and images for: Effects of mineralocorticoid receptor antagonists in patients with preserved ejection fraction: a meta-analysis of randomized clinical trials
Source: BMC Med. 2015 Jan 19;13:10. doi: 10.1186/s12916-014-0261-8 (PMC4307751; doi:10.1186/s12916-014-0261-8)

# Figure S1

**A**

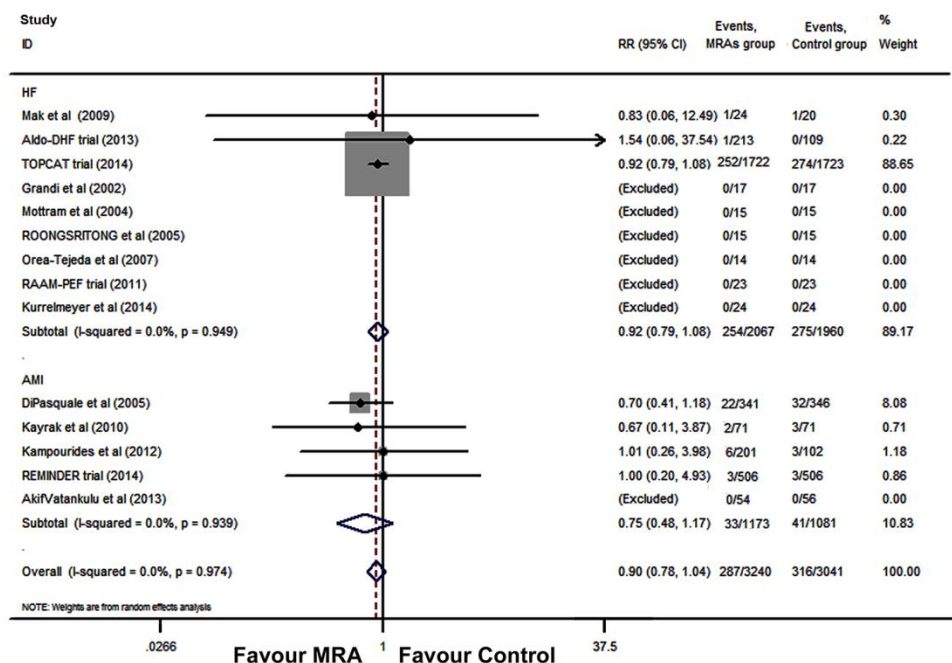

**B**

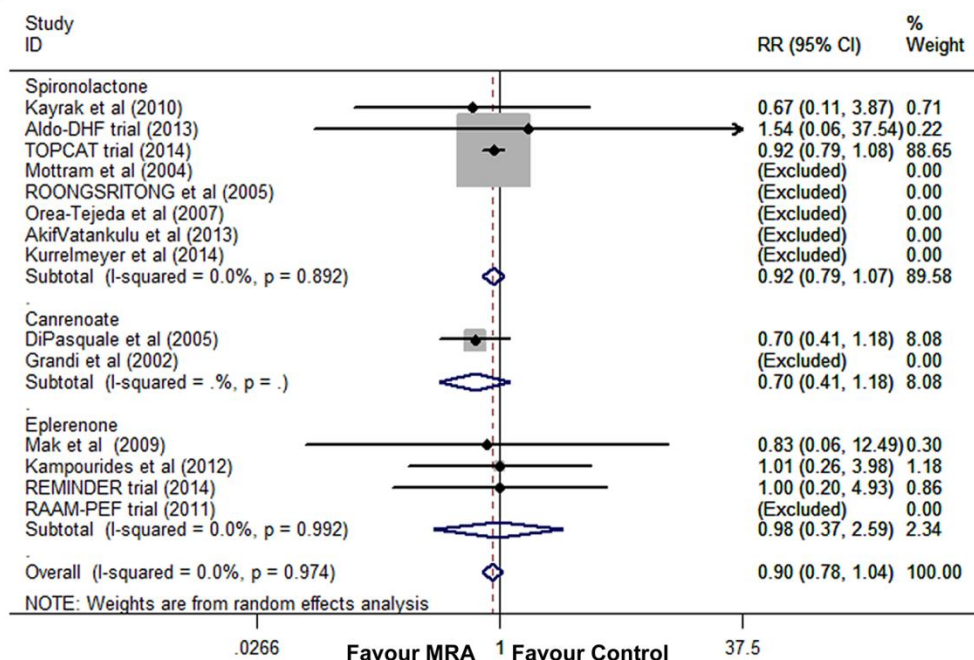

Figure S1

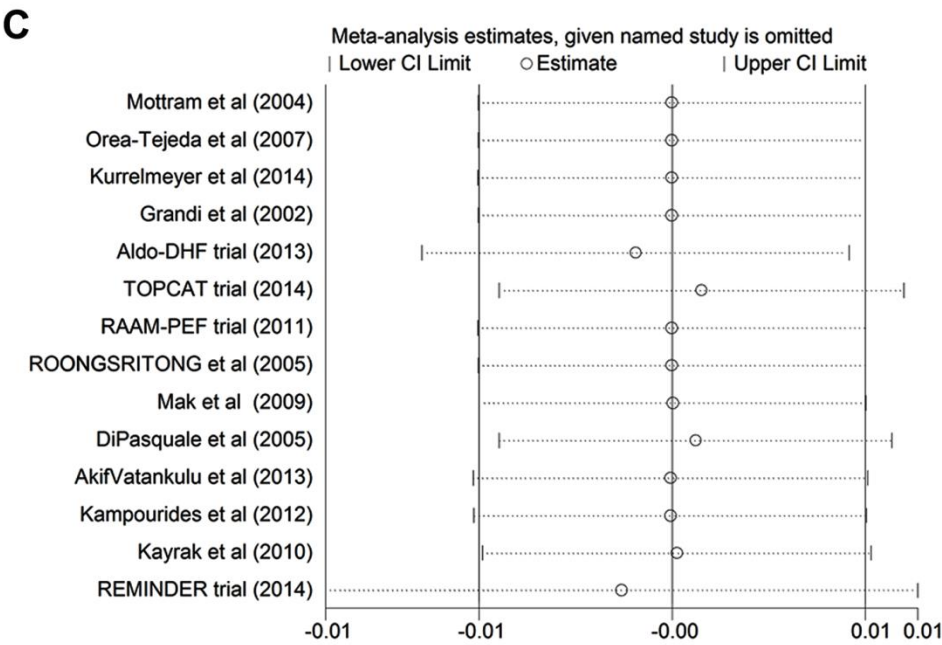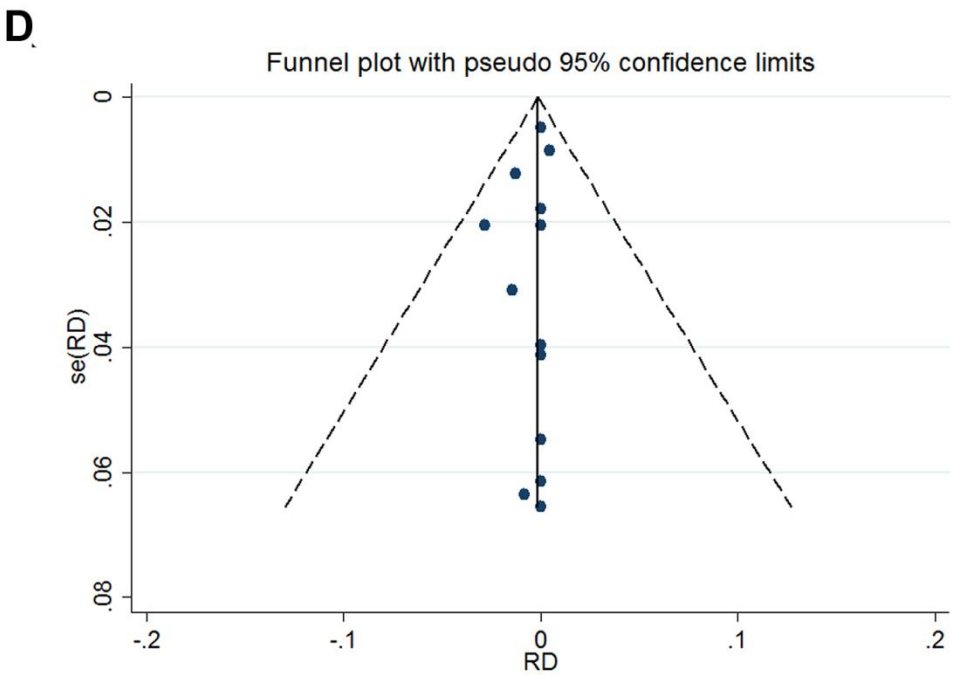

Figure S2

A

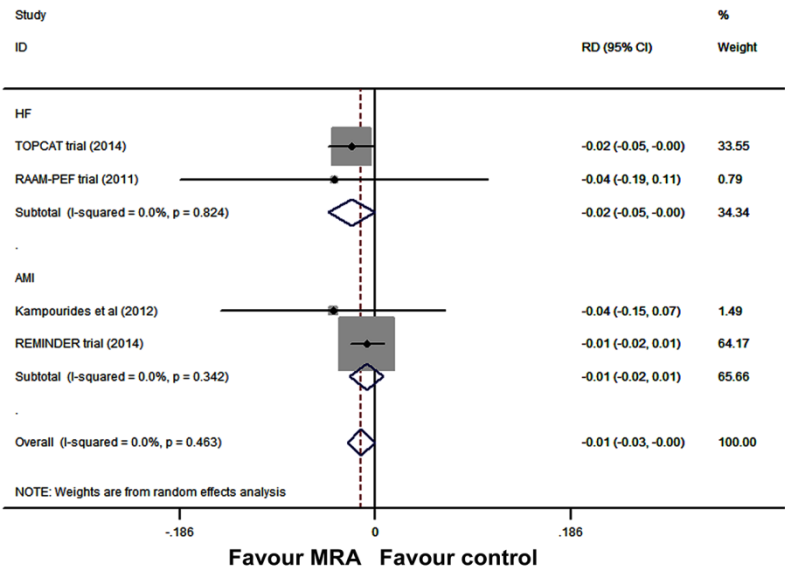

B

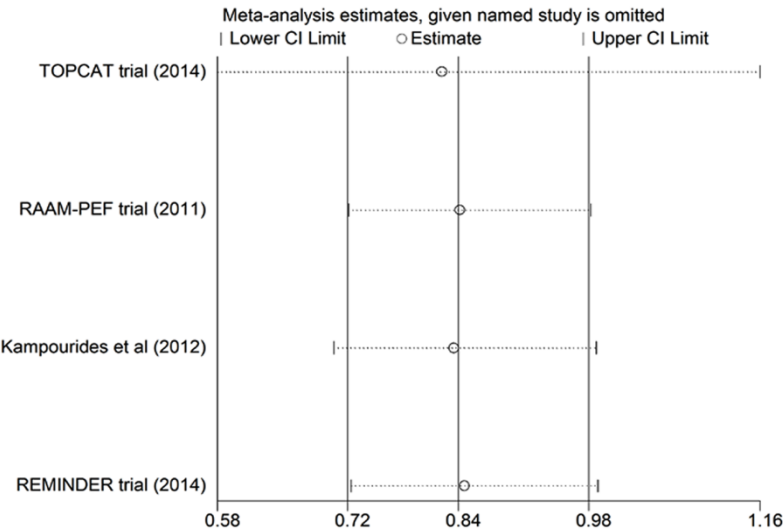

C

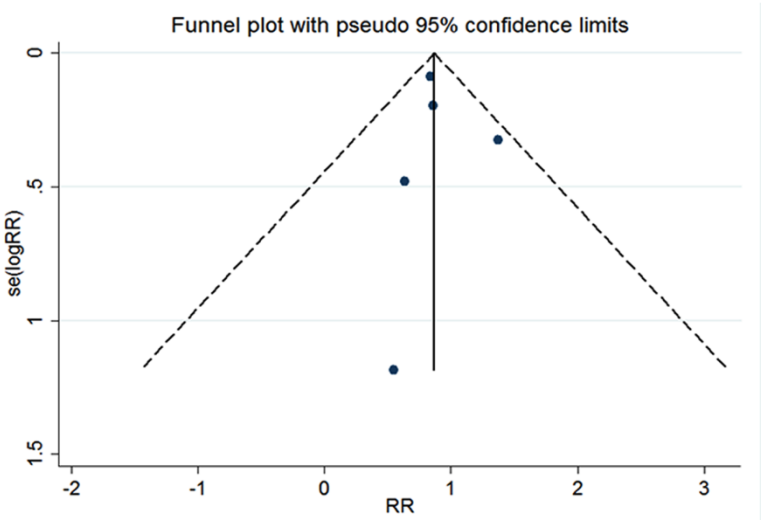

# Figure S3

**A**

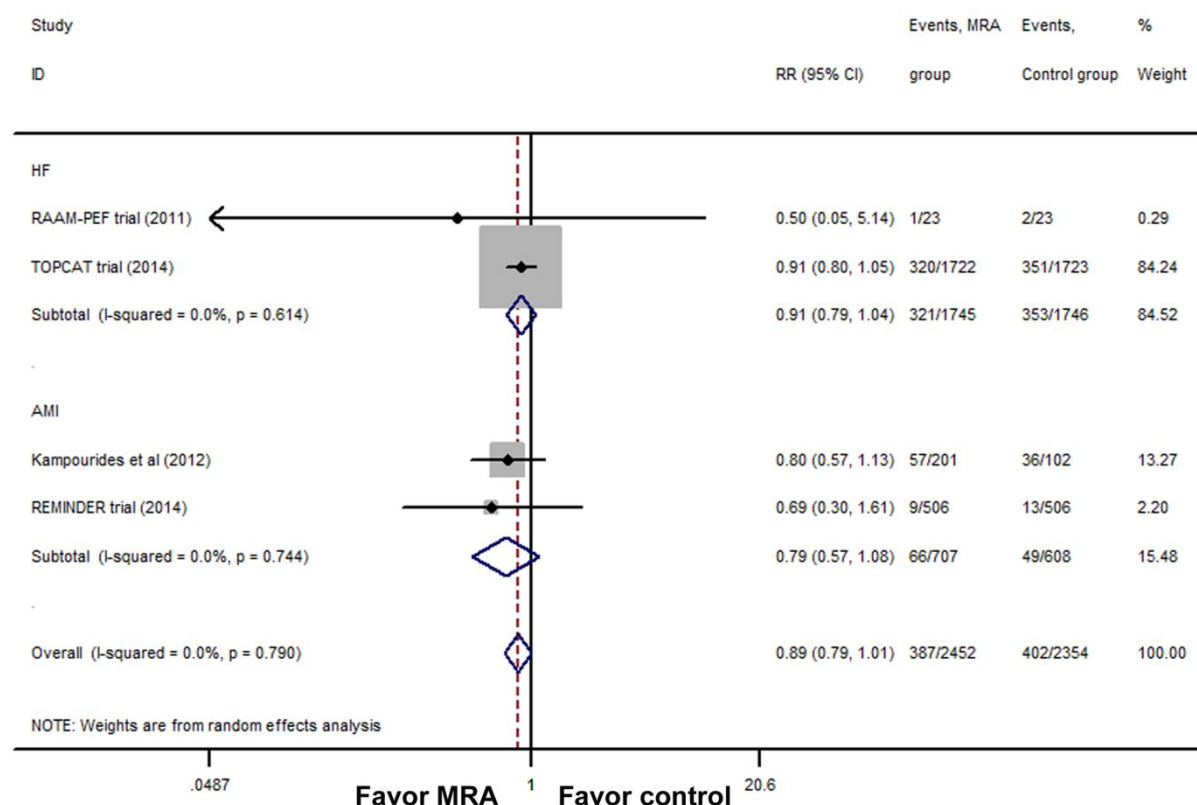

**B**

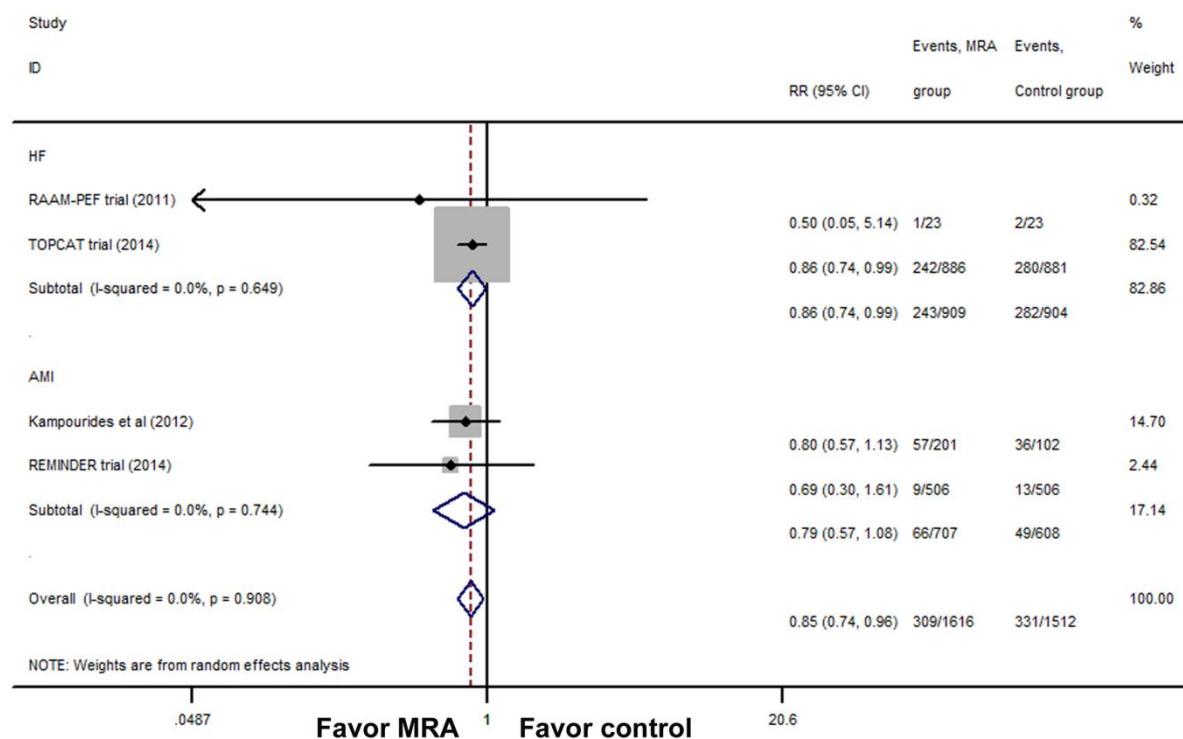

# Figure S4

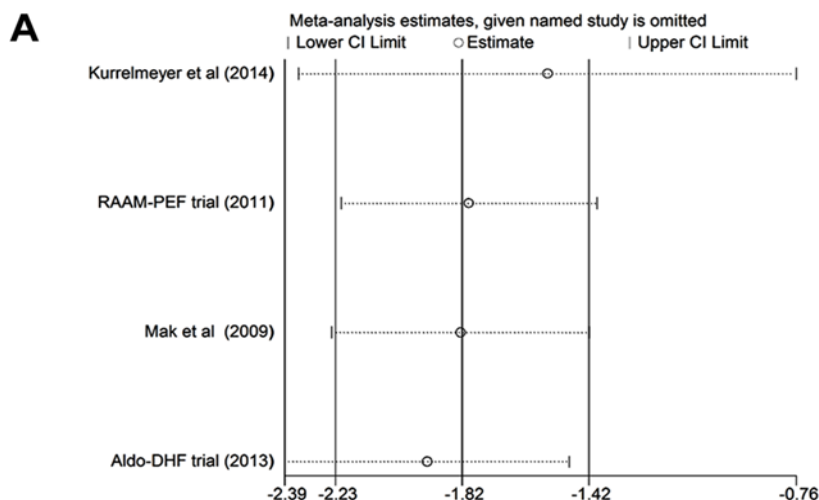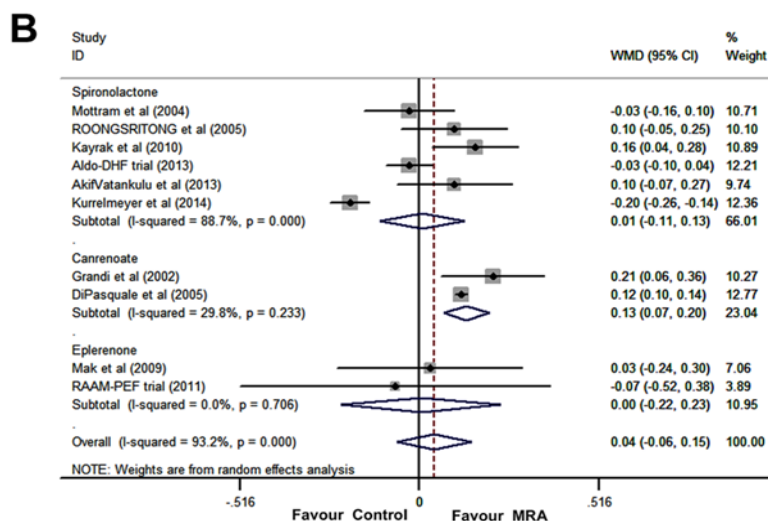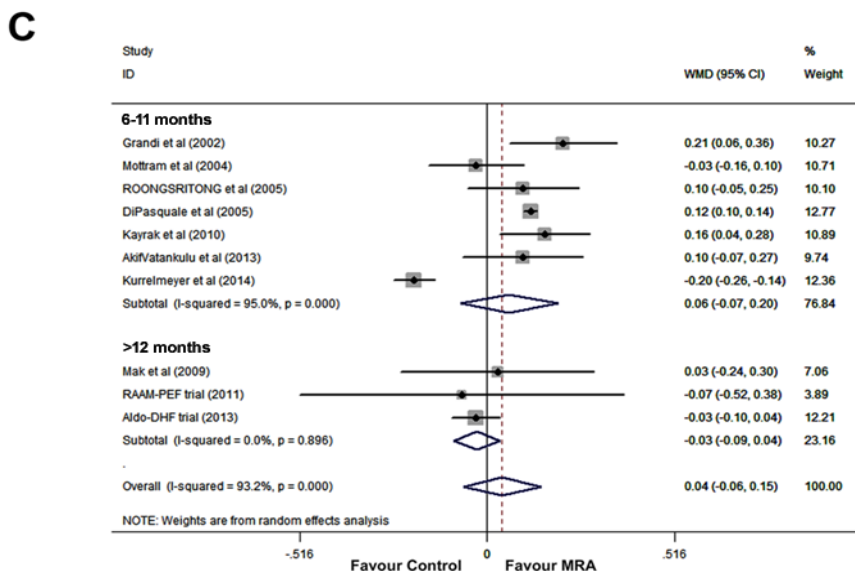

Figure S5 A

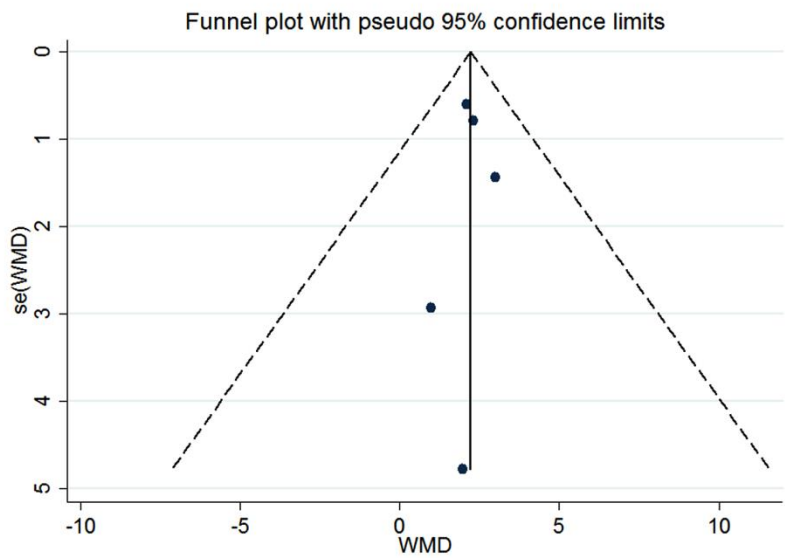

B

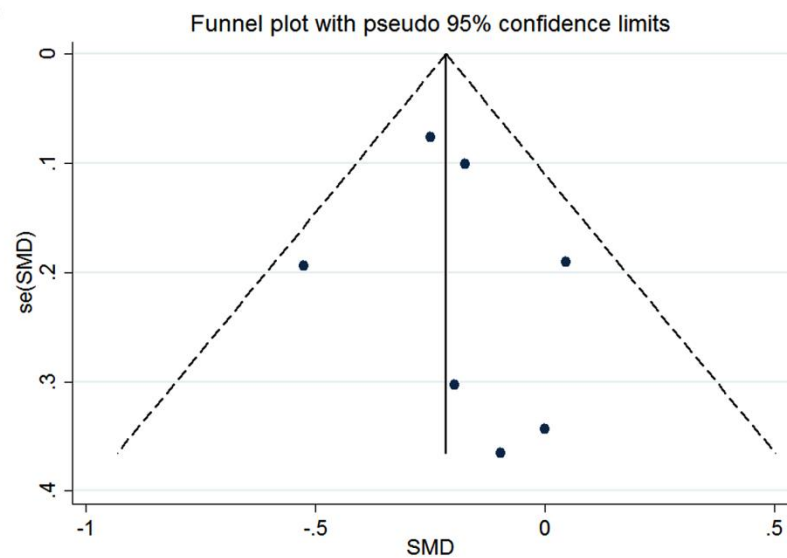

C

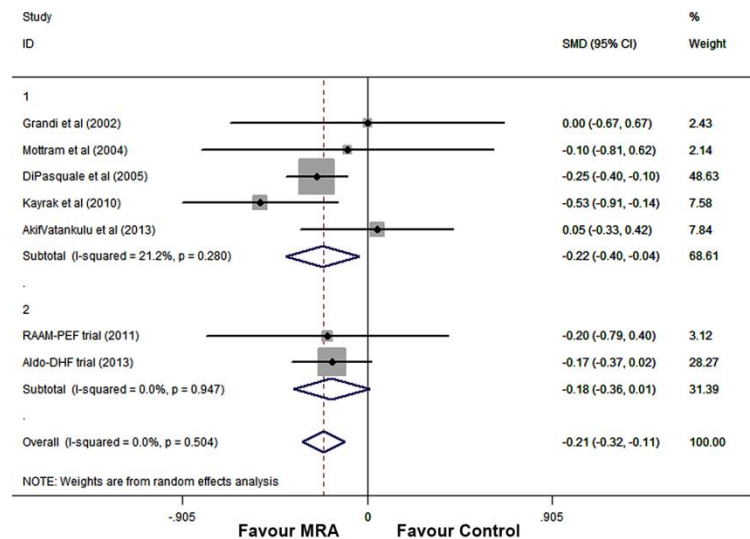

Supplement: Additional file 4: Figure S1. — Effect of MRAs on all-cause mortality. (A) Pooled effect estimate RR on all-cause mortality; (B) Subgroup analysis by drug type; (C) Leave-one-out analysis; (D) Funnel plots for all-cause mortality. CI, Confidence interval; MRA, Mineralocorticoid receptor antagonist; RR, Relative risk; SE, Standard error. Figure S2. Effect of MRAs on hospitalization for heart failure. (A) Pooled effect estimate RD on hospitalization for heart failure; (B) Leave-one-out analysis; (C)Funnel plots for hospitalization for heart failure. CI, Confidence interval; MRA, Mineralocorticoid receptor antagonist; RD, Absolute risk reduction; SE, Standard error. Figure S3. Effect of MRAs on composite outcomes for deaths due to cardiovascular causes, aborted cardiac arrest, or hospitalization due to heart failure. (A) Pooled effect estimate RR on composite outcomes; (B) Pooled analysis excluding patients randomized into TOPCAT trial from Russia and the Republic of Georgia on composite outcomes.CI, Confidence interval; MRA, Mineralocorticoid receptor antagonist; RR, Relative risk; SE, Standard error. Figure S4. Funnel plots and subgroup analyses of echo indexes of diastolic function. (A) Leave-one-out analysis for E/e'; (B) Subgroup analyses by drug type; (C) Subgroup analyses by treatment duration. CI, Confidence interval; E/A ratio, the ratio of early to late diastolic transmitral flow; E/e', an echocardiographic estimate of filling pressure for assessment of diastolic function; MRA, Mineralocorticoid receptor antagonist; WMD, Weighted mean difference. Figure S5. Subgroup analyses of indexes of cardiac structure and function. (A) Funnel plots for LVEF; (B) Subgroup analyses by LVEDD; (C) Subgroup analyses by treatment duration. LVEDD, Left ventricular end-diastolic diameter; LVEF, Left ventricular ejection fraction; LVMI, Left ventricular mass index; SMD, Standardized mean difference; MRA, Mineralocorticoid receptor antagonist; WMD, Weighted mean difference. [file 12916_2014_261_MOESM4_ESM.pdf]
